# Basic psychological need satisfaction and creativity among university students: the mediating role of intrinsic motivation

**DOI:** 10.3389/fpsyg.2026.1940043

**Published:** 2026-09-16

**Authors:** Rui Zhou, Che Aleha Ladin, Asbah Razali

**Affiliations:** 1 Institute for Advanced Studies, Universiti Malaya, Kuala Lumpur, Malaysia; 2 Department of Educational Foundations and Humanities, Faculty of Education, Universiti Malaya, Kuala Lumpur, Malaysia; 3 Department of Anthropology and Sociology, Faculty of Arts and Social Sciences, Universiti Malaya, Kuala Lumpur, Malaysia

**Keywords:** autonomy, competence, creative ideation, creativity, intrinsic motivation, relatedness

## Abstract

Creativity has received considerable attention as a core competency in higher education, yet the psychological processes associated with everyday creative ideation remain insufficiently understood. Drawing on self-determination theory and the componential theory of creativity, this study examined the relationships among autonomy, competence, relatedness, intrinsic motivation, and creativity, operationalized as self-reported everyday creative ideation, in a sample of 1,067 Chinese undergraduate students. Structural equation modeling was employed, with age and gender included as control variables in the structural model. The results showed that autonomy, competence, and relatedness were all significantly and positively associated with intrinsic motivation (β = 0.299, *p* < 0.001; β = 0.218, *p* < 0.001; β = 0.370, *p* < 0.001, respectively), and intrinsic motivation was significantly and positively associated with everyday creative ideation (β = 0.252, *p* < 0.001). Autonomy, competence, and relatedness were also significantly and positively associated with everyday creative ideation (β = 0.252, *p* < 0.001; β = 0.209, *p* < 0.001; β = 0.198, *p* = 0.002, respectively). Further analyses revealed significant indirect relationships between autonomy, competence, and relatedness and everyday creative ideation through intrinsic motivation (β = 0.075, *p* = 0.002; β = 0.055, *p* = 0.004; β = 0.093, *p* = 0.002, respectively). Simulation-based sensitivity analyses further indicated that these indirect relationships remained relatively robust under moderate levels of potential unmeasured confounding. These findings provide further theoretical insight into the associations among basic psychological need satisfaction, intrinsic motivation, and creativity in higher education, while the practical implications are primarily relevant to supporting students’ creative ideation.

## 1 Introduction

Against the backdrop of rapid technological advancement and accelerating globalization, higher education plays an important role in developing highly qualified and creative talent to support national innovation strategies (Li and Xu, 2025). Creativity is a key competency that enables university students to address complex problems (Jiang et al., 2025). Empirical research has shown that creative learning environments are positively associated with creativity among university students (Fan and Cai, 2022).

The development of creativity is closely related to the interplay between cognitive resources and affective experiences. In terms of cognitive resources, knowledge integration ability (Adeel et al., 2023), cognitive flexibility (Preiss, 2022), and problem-solving ability (Alt et al., 2023) have all been linked to students’ creative performance. Among motivational and affective factors, emotion regulation, intrinsic motivation, autonomy, competence, and relatedness play important roles in students’ sustained engagement and exploratory behavior during creative activities (Ladas et al., 2024; Yesuf et al., 2023; Mammadov et al., 2025). However, existing research has focused more extensively on cognitive resources related to creativity, while systematic examination of the interplay among motivational and affective factors remains limited.

Self-determination theory (SDT) posits that autonomy, competence, and relatedness are basic psychological needs essential to individuals’ psychological growth and motivational development (Deci and Ryan, 2000). Satisfaction of these basic psychological needs can support students in realizing their creative potential and generating novel ideas (Weng et al., 2025). Previous research has shown that students’ perceived autonomy, competence, and relatedness are significantly associated with creativity (Singh and Aziz, 2025). Similarly, intrinsic motivation refers to engaging in an activity for its inherent interest and satisfaction. It has been associated with writing skills and online creativity (Alzubi and Nazim, 2024; Wang, 2022). Componential Theory of Creativity (CTC) further emphasizes the important role of intrinsic motivation in creativity (Amabile, 2013). Students’ perceived autonomy, competence, and relatedness have also been found to be significantly associated with intrinsic motivation (Loopers et al., 2024).

Although previous studies have separately examined relationships involving autonomy, competence, relatedness, intrinsic motivation, and creativity (Li et al., 2024; Putney et al., 2024; Jnaneswar and Ranjit, 2022), research remains limited on how these variables are jointly related within a unified theoretical framework. Recent empirical advances suggest that examining these interconnected pathways may provide a more comprehensive understanding of the complex processes associated with creativity development among university students within higher education (Li et al., 2025; Urban et al., 2025).

Accordingly, this study focuses on Chinese undergraduate students and examines the relationships of autonomy, competence, and relatedness with creativity, as well as the mediating role of intrinsic motivation. Drawing on self-determination theory and the componential theory of creativity, this study proposes a theoretically guided mediation model to examine the pathways linking these variables. Given the cross-sectional design, the study is not intended to support direct causal inference but rather to examine the statistical associations and indirect relationships among the variables specified in the theoretical model.

Creativity refers to the ability to produce outcomes that are both novel and appropriate (Sternberg and Lubart, 1996). Originality and usefulness are two important characteristics of creativity (Mayer, 1999). Researchers have defined creativity in somewhat different ways. Wallas (1926) conceptualized creativity as a multistage process involving preparation, incubation, illumination, and verification. Torrance (1988) described creativity as a sequence of processes involving problem identification, speculation, hypothesis construction, creation, and the communication of ideas to others. Within the componential theory of creativity, Amabile (2013) defined creativity as the ability to generate novel and appropriate responses, products, or solutions to open-ended tasks. Kaufman and Beghetto (2009) further argued that creativity is not limited to a small number of individuals with exceptional achievements but is also evident in everyday learning and daily activities.

Creativity is widely present in everyday life, and assessing individual creativity therefore requires attention to everyday creative expression (Richards, 2007). The Runco Ideational Behavior Scale developed by Runco et al. (2001) primarily assesses individuals’ tendencies to generate ideas in daily life. Kaufman’s (2012) Kaufman Domains of Creativity Scale evaluates individuals’ perceptions of their own creativity across five domains: everyday, scholarly, performance, mechanical or scientific, and artistic creativity. Fields and Bisschoff (2014) developed a comprehensive assessment instrument specifically for university students.

According to self-determination theory, autonomy, competence, and relatedness are regarded as three basic psychological needs (Deci and Ryan, 2000) and are closely associated with positive learning behaviors such as students’ active participation, exploration, and expression of ideas. A growing body of research has shown that basic psychological need satisfaction is significantly associated with students’ learning engagement (Nguyen-Viet and Nguyen-Viet, 2025), academic achievement (Zupančič et al., 2024), and creativity (Singh and Aziz, 2025).

Autonomy refers to the extent to which individuals experience their behavior as congruent with their sense of self (Ryan and Deci, 2020). Individuals with greater autonomy tend to perceive their activities as driven by personal interest or voluntary choice rather than by external control, and greater autonomy has been positively associated with creative thinking (Orakci and Durnali, 2023). Previous research has consistently shown a positive relationship between autonomy and creativity. For example, in workplace settings, individuals with greater autonomy tend to exhibit higher levels of creativity (Alhassan et al., 2022). Greater perceived autonomy has also been found to foster creativity (Li et al., 2018). Beyond these direct relationships, previous studies have suggested that the relationship between autonomy and creativity may also operate through vitality and supervisor support (Yu et al., 2021; Kivrak et al., 2025). However, the indirect relationship between autonomy and creativity through intrinsic motivation remains insufficiently examined. To further understand this relationship, the present study draws on self-determination theory to incorporate intrinsic motivation into a theoretical model linking autonomy and creativity and to examine whether autonomy is indirectly associated with creativity through intrinsic motivation.

Competence refers to the psychological experience of feeling capable of meeting challenges and successfully completing tasks (Ryan and Deci, 2020). It has also been shown to positively influence students’ creativity (Singh and Aziz, 2025). Empirical research has consistently shown that satisfaction of the need for competence can strengthen individuals’ self-confidence and promote persistence in creative activities (Ye et al., 2025). It has also been positively associated with learning engagement (Wang et al., 2021) and with greater efficiency and creativity in seeking solutions to challenging problems (Pitafi and Xie, 2024). In educational settings, competence provides university students with an important psychological foundation for actively approaching challenges and maintaining confidence, both of which are important for creative performance. The present study further examines whether competence is indirectly associated with creativity through intrinsic motivation.

Relatedness refers to the psychological state in which individuals feel positively connected to others and experience a sense of being respected and accepted (Ryan and Deci, 2020). Relatedness emphasizes the quality of university students’ relationships rather than merely the quantity of interpersonal contact. Students whose need for relatedness is satisfied may experience greater interpersonal support during participation, which can influence their creative tendencies (Singh and Aziz, 2025). Their level of engagement has also been positively associated with creative self-concept (Álvarez-Huerta et al., 2021). Relatedness can therefore be regarded as an important psychological factor associated with creativity among university students. The present study further examines whether relatedness is indirectly associated with creativity through intrinsic motivation.

According to self-determination theory, intrinsic motivation refers to engaging in an activity for its inherent interest and satisfaction rather than for external outcomes (Ryan and Deci, 2000). For university students, intrinsic motivation is reflected in their voluntary engagement in activities that they find inherently interesting and appealing. Autonomy and competence are essential for the development and maintenance of intrinsic motivation (Vansteenkiste et al., 2020), while positive feedback can enhance individuals’ interest in and enjoyment of an activity. Students with a stronger sense of competence are more likely to engage in activities voluntarily and persistently (Singh and Aziz, 2025), and competence is closely associated with intrinsic motivation (Bureau et al., 2022). Although the relative importance of autonomy may vary across learning contexts, satisfaction of the needs for competence and relatedness has likewise been associated with higher levels of intrinsic motivation (Hosseini et al., 2022; Loopers et al., 2024).

In the componential theory of creativity, intrinsic motivation is considered a key factor in creativity, and individuals are more likely to exhibit creative behavior when they are primarily motivated by the interest and challenge inherent in a task (Amabile, 2013). Studies across different educational stages have identified a relatively stable relationship between intrinsic motivation and creativity (Zhang et al., 2024; Li et al., 2025). At the university level, students are generally expected to assume greater responsibility for self-directed learning and self-management (Fazey and Fazey, 2001). Intrinsic motivation has been identified as a key driving force behind university students’ creativity (Ma et al., 2023) and can encourage greater engagement and creativity in their academic work (Gulzar et al., 2022). Accordingly, the present framework examines not only the direct relationship between intrinsic motivation and creativity but also whether autonomy, competence, and relatedness are indirectly associated with creativity through intrinsic motivation.

The literature suggests that, although previous studies have established a positive relationship between motivation and creativity, the relationship between intrinsic motivation and creativity among university students warrants further examination in higher education contexts (Kreitler and Casakin, 2009; Pan et al., 2023). The componential theory of creativity proposes that individuals are most creative when they are motivated by the inherent interest of the task, highlighting the important role of intrinsic motivation (Amabile et al., 1996). In addition, the relationships of autonomy, competence, and relatedness with creativity require further clarification. Based on self-determination theory (Ryan and Deci, 2000), autonomy, competence, and relatedness provide important psychological conditions for the development of intrinsic motivation and are associated with positive developmental outcomes, including creativity. Intrinsic motivation may therefore play an indirect role in these associations. Specifically, students with higher levels of autonomy, competence, and relatedness may demonstrate higher levels of intrinsic motivation, which in turn is associated with greater creativity. Previous studies have shown that these three basic psychological needs and intrinsic motivation are positively associated with creativity-related outcomes (Urban et al., 2025; Mammadov et al., 2025; Yesuf et al., 2023). Taken together, these theoretical and empirical considerations provide the basis for the following hypotheses.

H1: Autonomy (H1a), competence (H1b), and relatedness (H1c) are positively associated with creativity among university students in China.

H2: Autonomy (H2a), competence (H2b), and relatedness (H2c) are indirectly associated with creativity through intrinsic motivation among university students in China.

Figure 1 presents the proposed theoretical model. The model posits that autonomy, competence, and relatedness may be directly associated with creativity and may also be indirectly associated with creativity through intrinsic motivation.

**Figure 1:**
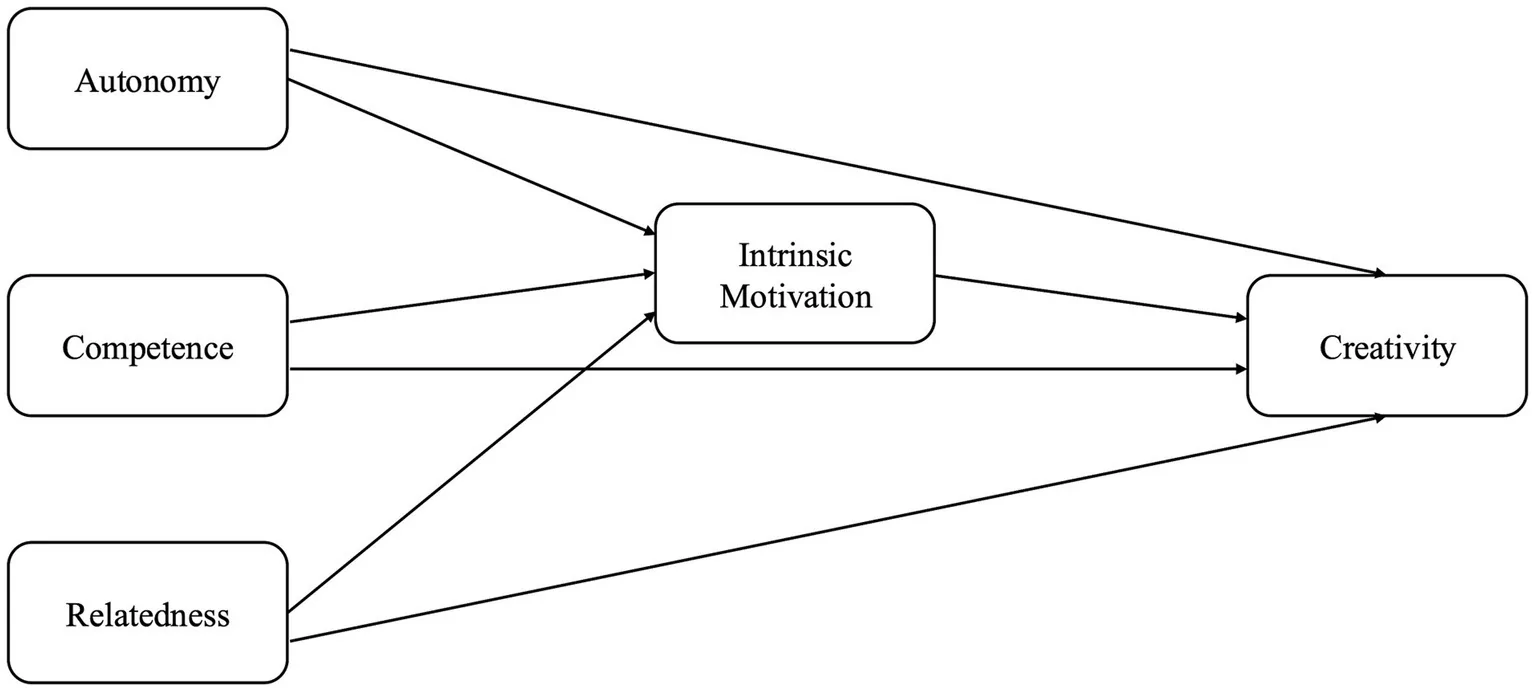
Research model.

## 2 Materials and methods

### 2.1 Participants and procedure

This study was conducted at five public universities in Henan Province, China. These institutions were selected for three main reasons. First, they represent different institutional tiers, including one university included in Project 211, a national higher education development initiative; one university included in the Double First-Class initiative; and three regular provincial universities. Second, they reflect institutional diversity, including comprehensive, normal, and science and engineering universities. This composition provided some diversity in institutional characteristics within the sample but should not be interpreted as a probability-based or representative sample of public universities in China. Full-time undergraduate students aged 18 to 23 were selected as the target population because this age range typically spans the transition from adolescence to adulthood, an important period of psychological development and a stage in which creativity can play an increasingly important role in students’ academic and career development.

The study protocol was approved by the Universiti Malaya Research Ethics Committee (UM.TNC(P&I)/UMREC/3/4651). Before formal data collection, participants were provided with an informed consent form explaining the purpose of the study, data confidentiality and anonymity, and their right to withdraw from the study at any time without providing a reason.

Data were collected through an online survey using convenience sampling between December 2025 and April 2026. All measurement instruments underwent a standard translation and back-translation procedure. The translation and back-translation were conducted by one professor and three researchers specializing in educational psychology. The original and back-translated versions were compared, and semantic discrepancies were resolved through discussion to ensure that the items retained the same conceptual meaning as the original scales. Before the formal survey, the adapted items were pilot tested with 20 undergraduate students who were not included in the main sample. Participants evaluated the clarity and comprehensibility of the item wording. The feedback indicated that the items were generally understandable and that no substantial comprehension difficulties were identified. This pilot test was intended to assess linguistic clarity rather than to establish the factorial validity of the adapted scale. For the main survey, approximately 1,300 questionnaires were distributed, and 1,287 responses were collected. Invalid responses were excluded during the data screening process, resulting in 1,067 valid responses for analysis. The age and gender distributions of the sample are presented in Table 1.

**Table 1:** Distribution of sample by age and gender.

Age	Male	Female	Total
18 years old	89	67	156
19 years old	149	134	283
20 years old	118	153	271
21 years old	73	72	145
22 years old	74	116	190
23 years old	10	12	22
Total	513	554	1,067

### 2.2 Measures

#### 2.2.1 Autonomy, competence, and relatedness

The Basic Psychological Need Satisfaction Scale (BPNS; Deci and Ryan, 2000; Gagné, 2003) was used to assess basic psychological need satisfaction among undergraduate students. The scale consists of 21 items assessing the extent to which individuals’ basic psychological needs are satisfied in daily life. It comprises three subscales: autonomy, competence, and relatedness, with 7, 6, and 8 items, respectively. In the present study, scores for the three subscales were calculated separately to reflect the extent to which each of the three basic psychological needs was satisfied. The original instrument uses a seven-point response format and includes both positively and negatively worded items. For the present study, the response format was changed to a five-point scale, and the negatively worded items were rephrased as positive statements to improve linguistic clarity and comprehensibility in Chinese. The complete English and Chinese wording of all 21 adapted items is provided in Supplementary Table. Previous studies have demonstrated good reliability and validity for the BPNS, and it has been widely used to assess basic psychological need satisfaction (Gagné, 2003; Shin and Park, 2022). In the present study, the three subscales showed satisfactory internal consistency (α = 0.781 for autonomy, α = 0.743 for competence, and α = 0.835 for relatedness).

#### 2.2.2 Intrinsic motivation

The Intrinsic Motivation Inventory (IMI; Ryan, 1982) was used to assess university students’ intrinsic motivation in creative activities. The IMI allows researchers to select appropriate subscales based on the aims of the study (Ryan, 1982). In the present study, the Interest/Enjoyment subscale was used with a minor adaptation, in which the word “activity” was replaced with “creative activity.” The subscale consists of 7 items rated on a five-point Likert scale ranging from 1 = “not at all true” to 5 = “very true,” with higher scores indicating greater interest and enjoyment in creative activities. The subscale showed good internal consistency in the present study (α = 0.856). Previous research has also provided evidence of good reliability and validity for the IMI among university students (Bosch, 2024).

#### 2.2.3 Creativity

The Runco Ideational Behavior Scale (RIBS; Runco et al., 2001) was used to assess university students’ self-reported everyday ideational behavior and related tendencies rather than objective creative performance. The scale consists of 23 items rated on a five-point Likert scale ranging from 1 = “never” to 5 = “very often.” It assesses two dimensions of ideational behavior. The first factor comprises 17 items and primarily reflects individuals’ tendency to generate and express creative ideas, whereas the second factor comprises 6 items and primarily reflects frequent idea generation and heightened ideational activity. In the present study, the scale demonstrated good internal consistency (α = 0.908 for the total scale, α = 0.887 for First Factor, and α = 0.822 for Second Factor). Previous studies using Chinese university student samples have also provided support for the internal consistency and structural validity of the RIBS (Xu et al., 2022; Sun et al., 2023).

#### 2.2.4 Control variables

Previous studies have suggested that individual creativity may vary by demographic characteristics such as gender and age (Park et al., 2023; Ross et al., 2023; Soler Pastor et al., 2022). Therefore, gender and age were included as control variables in the structural model.

### 2.3 Data analysis

To examine the structural relationships among autonomy, competence, relatedness, intrinsic motivation, and creativity, this study employed a multistep data analysis approach. Data analyses were conducted using IBM SPSS Statistics 27 and SmartPLS 4.1.3. Given the cross-sectional nature of the data, the analyses aimed to examine the structural associations among the variables rather than infer causal relationships.

Means, standard deviations, skewness, and kurtosis were first calculated to describe the distributions of the main variables. Independent-samples t tests and one-way analyses of variance (ANOVA) were then conducted to compare differences in the study variables across gender and age groups. Finally, Pearson correlation analysis was used to examine the direction and strength of the relationships among the constructs.

This study employed partial least squares structural equation modeling (PLS-SEM) to estimate the measurement and structural models. Although the present study was theoretically driven and proposed and tested hypothesized relationships among the variables, the analytical objectives were not limited to examining the significance of structural paths but also included evaluating the explained variance of key endogenous constructs and the predictive relevance of the structural model. PLS-SEM emphasizes maximizing the explained variance of endogenous constructs and is particularly suitable for research focusing on variance explanation, predictive relevance, and the evaluation of complex structural relationships (Hair et al., 2022). Furthermore, creativity was specified as a reflective–reflective higher-order construct comprising two reflective first-order dimensions, namely, First Factor and Second Factor. A two-stage approach was employed. In the first stage, the two first-order dimensions were estimated using their respective reflective indicators. In the second stage, the latent variable scores of First Factor and Second Factor were used as reflective indicators of the higher-order creativity construct (Sarstedt et al., 2019).

Model evaluation was conducted following the recommendations of Hair et al. (2019, 2021). First, the reflective measurement models were assessed in terms of indicator reliability, internal consistency reliability, convergent validity, and discriminant validity. Indicator reliability was evaluated using outer loadings, with 0.708 used as the reference criterion. Internal consistency reliability was assessed using Cronbach’s alpha (α ≥ 0.70) and composite reliability (CR ≥ 0.70), convergent validity was assessed using average variance extracted (AVE ≥ 0.50), and discriminant validity was evaluated using the heterotrait–monotrait ratio (HTMT < 0.85). For the adapted BPNS, the initial measurement model included all 21 administered items. Items with outer loadings below 0.708 were not removed automatically. Item-retention decisions were based jointly on the magnitude of the outer loading, the effect of item removal on CR and AVE, the conceptual correspondence between each item and its intended psychological need, potential content redundancy, and the adequacy of the remaining content coverage. Thus, statistical and substantive considerations were evaluated together. Following this assessment, six BPNS items were removed, and the measurement model was re-estimated. Subsequently, age and gender were included in the model as control variables, and the structural model was evaluated. The main evaluation criteria included standardized path coefficients (β), bootstrap-based statistical significance and confidence intervals, variance inflation factors (VIF) for assessing multicollinearity, effect sizes (f^2^), explained variance (R^2^ and adjusted R^2^), out-of-sample predictive performance assessed using PLSpredict with 10 folds and 10 repetitions ( $Q_{\text{predict}}^{2}$ ), and the standardized root mean square residual (SRMR) as an approximate model fit index. The direct and indirect effects in the hypothesized model were tested using 5,000 bootstrap resamples. An effect was considered statistically significant when its 95% confidence interval did not include zero. Given the cross-sectional nature of the data, these results were interpreted only as statistical associations among the variables within the hypothesized model rather than as evidence of causal relationships (Maxwell and Cole, 2007).

Because the mediation model was estimated using cross-sectional observational data, the indirect effects may be affected by potential unmeasured confounding. To assess the sensitivity of the mediation results to potential unmeasured confounding, this study employed the mediation sensitivity analysis approach proposed by Tingley et al. (2014). Sensitivity analyses were conducted separately for the three mediation pathways using R version 4.5.0 with the mediation package (version 4.5.1), with age and gender controlled for in both the mediator and outcome models. This approach uses the correlation coefficient ρ between the residuals of the mediator and outcome models as the sensitivity parameter, where ρ = 0 indicates that the residuals of the two models are uncorrelated. Higher assumed values of ρ represent stronger residual correlation potentially attributable to unmeasured common influences. In this study, ρ was varied in increments of 0.01, and the corresponding indirect effects were re-estimated under different values of ρ to assess the sensitivity of the indirect effect estimates to potential unmeasured confounding. The analysis used 1,000 simulations, and the value of ρ at which the estimated indirect effect decreased to zero was taken as the sensitivity threshold. This threshold reflects the degree of residual correlation between the mediator and outcome models required, within the specified sensitivity analysis framework, for the estimated indirect effect to decrease to zero. This analysis provides additional evidence for assessing the stability of the observed indirect relationships under potential unmeasured confounding.

## 3 Results

### 3.1 Common method bias test

Because this study collected data using self-report questionnaires, the relationships among the variables may be affected by common method bias (Podsakoff et al., 2003). To examine this issue, Harman’s single-factor test was conducted following the recommendation of Zhou and Long (2004). All measurement items as administered were entered into an unrotated exploratory factor analysis. The results showed that the first factor accounted for 35.439% of the total variance, which was below the commonly used 40% threshold.

Following item refinement, common method bias was further assessed through a full collinearity assessment within the PLS-SEM framework, as recommended by Kock (2015). The variance inflation factor (VIF) values for autonomy, competence, relatedness, intrinsic motivation, and creativity were 2.384, 1.830, 2.274, 2.550, and 2.472, respectively. All values were below the recommended threshold of 3.3, further indicating that the data were not substantially affected by common method bias.

### 3.2 Descriptive statistics

In this section and subsequent analyses, creativity refers to self-reported everyday creative ideation as measured by the Runco Ideational Behavior Scale (RIBS). Table 2 presents the descriptive statistics for autonomy, competence, relatedness, intrinsic motivation, and creativity. The means of the five variables ranged from 3.415 to 3.568. Relatedness had the highest mean score (M = 3.568, SD = 0.741), followed by competence (M = 3.557, SD = 0.726) and intrinsic motivation (M = 3.468, SD = 0.787). In comparison, everyday creative ideation (M = 3.423, SD = 0.684) and autonomy (M = 3.415, SD = 0.760) had slightly lower mean scores. The skewness and kurtosis results indicated slight negative skewness across the five variables. Specifically, autonomy (skewness = −0.989, kurtosis = 0.451), competence (skewness = −0.844, kurtosis = 0.587), relatedness (skewness = −0.973, kurtosis = 0.675), intrinsic motivation (skewness = −1.005, kurtosis = 0.395), and everyday creative ideation (skewness = −1.111, kurtosis = 0.804) all had skewness and kurtosis values within acceptable ranges (|skewness| < 3; |kurtosis| < 10), and none of the variables showed severe univariate non-normality (Kline, 2016).

**Table 2:** Descriptive statistics and normality assessment of variables.

Variable	Minimum	Maximum	Mean	SD	Skewness	Kurtosis
Autonomy	1.000	5.000	3.415	0.760	−0.989	0.451
Competence	1.000	5.000	3.557	0.726	−0.844	0.587
Relatedness	1.000	5.000	3.568	0.741	−0.973	0.675
Intrinsic motivation	1.000	5.000	3.468	0.787	−1.005	0.395
Creativity	1.000	5.000	3.423	0.684	−1.111	0.804

### 3.3 Analysis of gender and age differences

As shown in Table 3, the independent-samples t tests indicated no statistically significant gender differences in autonomy (t = −0.754, *p* = 0.451), competence (t = 0.569, *p* = 0.569), relatedness (t = 0.994, *p* = 0.321), intrinsic motivation (t = 0.380, *p* = 0.704), or creativity (t = 1.091, *p* = 0.275). Thus, no significant gender differences were observed across the core study variables.

**Table 3:** Gender differences in study variables.

Variable	Gender	*N*	Mean	SD	t-value	*p*-value
Autonomy	Male	513	3.396	0.780	−0.754	0.451
	Female	554	3.431	0.742		
Competence	Male	513	3.570	0.747	0.569	0.569
	Female	554	3.545	0.707		
Relatedness	Male	513	3.591	0.722	0.994	0.321
	Female	554	3.546	0.757		
Intrinsic motivation	Male	513	3.478	0.774	0.380	0.704
	Female	554	3.459	0.799		
Creativity	Male	513	3.446	0.660	1.091	0.275
	Female	554	3.401	0.706		

As shown in Table 4, the one-way ANOVA results indicated significant differences across age groups in competence (*F* = 3.437, *p* = 0.004), relatedness (*F* = 3.751, *p* = 0.002), and creativity (*F* = 2.736, *p* = 0.018). In contrast, no significant differences across age groups were observed for autonomy (*F* = 1.852, *p* = 0.100) or intrinsic motivation (*F* = 0.862, *p* = 0.506). The mean scores of the variables did not show a consistent increasing or decreasing trend with age, and the observed age-group differences were primarily evident in competence, relatedness, and creativity.

**Table 4:** Age differences in the study variables.

Variable	Age	*N*	Mean	SD	*F*-value	*p*-value
Autonomy	18	156	3.502	0.734	1.852	0.100
	19	283	3.475	0.786		
	20	271	3.336	0.803		
	21	145	3.350	0.741		
	22	190	3.393	0.674		
	23	22	3.597	0.823		
Competence	18	156	3.623	0.720	3.437	0.004
	19	283	3.673	0.613		
	20	271	3.498	0.793		
	21	145	3.493	0.782		
	22	190	3.445	0.691		
	23	22	3.720	0.952		
Relatedness	18	156	3.501	0.760	3.751	0.002
	19	283	3.694	0.687		
	20	271	3.480	0.799		
	21	145	3.494	0.747		
	22	190	3.581	0.662		
	23	22	3.869	0.884		
Intrinsic motivation	18	156	3.480	0.780	0.862	0.506
	19	283	3.519	0.791		
	20	271	3.426	0.808		
	21	145	3.399	0.763		
	22	190	3.474	0.736		
	23	22	3.649	1.078		
Creativity	18	156	3.444	0.755	2.736	0.018
	19	283	3.513	0.629		
	20	271	3.304	0.727		
	21	145	3.453	0.648		
	22	190	3.423	0.641		
	23	22	3.372	0.759		

### 3.4 Correlation analysis

As shown in Table 5, the Pearson correlation analysis indicated that autonomy (*r* = 0.730), competence (*r* = 0.677), and relatedness (*r* = 0.697) were all significantly and positively correlated with everyday creative ideation. In addition, autonomy (*r* = 0.682), competence (*r* = 0.630), and relatedness (*r* = 0.705) were all significantly and positively correlated with intrinsic motivation, and intrinsic motivation was also significantly and positively correlated with everyday creative ideation (*r* = 0.709). These results provided a preliminary basis for the subsequent structural model analysis and mediation analysis.

**Table 5:** Pearson correlation matrix.

Variable	AU	CO	RE	IM	CRE
AU	–				
CO	0.676 (<0.001)	–			
RE	0.674 (<0.001)	0.614 (<0.001)	–		
IM	0.682 (<0.001)	0.630 (<0.001)	0.705 (<0.001)	–	
CRE	0.730 (<0.001)	0.677 (<0.001)	0.697 (<0.001)	0.709 (<0.001)	–

AU, autonomy; CO, competence; RE, relatedness; IM, intrinsic motivation; CRE, everyday creative ideation (RIBS). Values in parentheses are two-tailed *p* values.

### 3.5 Measurement model evaluation

The initial BPNS measurement model included all 21 adapted items. Based on the statistical and content-evaluation criteria described above, six items were excluded, and the model was subsequently re-estimated using the remaining 15 items. The resulting 15-item set is therefore treated as a sample-specific refinement rather than as a recommended Chinese short form of the BPNS. The complete item-level diagnostic results, including the outer loadings from the initial and final models and the corresponding item-retention decisions, are reported in Supplementary Table.

Table 6 presents the results of the reliability and validity assessment for the first-order measurement model. The results showed that Cronbach’s alpha values for the constructs ranged from 0.743 to 0.887, while composite reliability values ranged from 0.838 to 0.909, indicating good internal consistency. The average variance extracted (AVE) values ranged from 0.527 to 0.585, supporting convergent validity. All HTMT values were below 0.85, indicating good discriminant validity among the constructs.

**Table 6:** First order measurement model evaluation.

Construct		Number of items	α	CR	AVE	HTMT					
						AU	CO	RE	IM	CRE	
										F1	F2
AU		5	0.781	0.851	0.534	–					
CO		4	0.743	0.838	0.565	0.763	–				
RE		6	0.835	0.879	0.548	0.807	0.675	–			
IM		7	0.856	0.891	0.538	0.816	0.738	0.807	–		
CRE	F1	17	0.887	0.909	0.527	0.803	0.771	0.745	0.772	–	
	F2	6	0.822	0.875	0.585	0.669	0.590	0.622	0.648	0.756	–

F1, First Factor; F2, Second Factor; CR, composite reliability; AVE, average variance extracted; HTMT, heterotrait-monotrait ratio.

The higher-order measurement model results for creativity are reported separately in Table 7. The Cronbach’s alpha value for higher-order creativity was 0.787, and the composite reliability (CR) was 0.903, indicating good internal consistency. The average variance extracted (AVE) was 0.823, supporting convergent validity. The HTMT values between higher-order creativity and the other main constructs ranged from 0.784 to 0.847, all below 0.85, supporting discriminant validity. The loadings of First Factor and Second Factor on the higher-order creativity construct were 0.929 and 0.884, respectively.

**Table 7:** Higher-order measurement model evaluation.

Construct	Number of dimensions	α	CR	AVE	HTMT				
					AU	CO	RE	IM	CRE
CRE	2	0.787	0.903	0.823	0.847	0.784	0.787	0.818	–

### 3.6 Structural model analysis

The model fit and multicollinearity of the structural model were first assessed. The SRMR was 0.060, below the conventional benchmark of 0.08 for approximate model fit in PLS-SEM (Henseler et al., 2016). The VIF values for the structural model ranged from 1.017 to 2.394, all below the threshold of 3.3, indicating no substantial multicollinearity concerns. Subsequently, the structural model paths were tested using a bootstrap procedure with 5,000 resamples, and age and gender were included as control variables in the structural model. The model explained 58.2% of the variance in intrinsic motivation (R^2^ = 0.582; adjusted R^2^ = 0.580) and 59.6% of the variance in creativity (R^2^ = 0.596; adjusted R^2^ = 0.594). PLSpredict yielded $Q_{\text{predict}}^{2}$ values of 0.572 for intrinsic motivation and 0.560 for creativity, both greater than zero, indicating out-of-sample predictive relevance for the two endogenous constructs (Hair et al., 2019). As shown in Table 8, the f^2^ values for the main structural paths ranged from 0.044 to 0.177. The relationship between relatedness and intrinsic motivation showed a medium effect size (f^2^ = 0.177), whereas the remaining main structural paths showed small effect sizes. Control-variable f^2^ values were ≤ 0.001.

**Table 8:** Structural model path coefficients and significance.

Path	β	t value	*p* value	95% Bootstrap CI	Effect size (f^2^)	Significance
AU → CRE	0.252	4.403	<0.001	[0.133, 0.358]	0.070	Significant
CO → CRE	0.209	4.970	<0.001	[0.126, 0.289]	0.062	Significant
RE → CRE	0.198	3.106	0.002	[0.068, 0.321]	0.044	Significant
AU → IM	0.299	5.857	<0.001	[0.190, 0.394]	0.106	Significant
CO → IM	0.218	5.007	<0.001	[0.131, 0.301]	0.070	Significant
RE → IM	0.370	6.199	<0.001	[0.254, 0.485]	0.177	Significant
IM → CRE	0.252	3.766	<0.001	[0.118, 0.379]	0.065	Significant
Age → CRE	0.023	1.086	0.278	[−0.018, 0.066]	0.001	Not significant
Age → IM	0.010	0.430	0.667	[−0.036, 0.055]	0.000	Not significant
Gender → CRE	−0.034	0.891	0.373	[−0.111, 0.040]	0.001	Not significant
Gender → IM	0.015	0.377	0.706	[−0.061, 0.095]	0.000	Not significant

CRE, everyday creative ideation (RIBS).

The path analysis results showed that all hypothesized structural paths in the model were statistically significant. Specifically, autonomy, competence, and relatedness were all significantly and positively associated with everyday creative ideation (β = 0.252, *p* < 0.001, 95% CI = [0.133, 0.358]; β = 0.209, *p* < 0.001, 95% CI = [0.126, 0.289]; β = 0.198, *p* = 0.002, 95% CI = [0.068, 0.321]), supporting H1. Autonomy, competence, and relatedness were also significantly and positively associated with intrinsic motivation (β = 0.299, *p* < 0.001; β = 0.218, *p* < 0.001; β = 0.370, *p* < 0.001). In addition, intrinsic motivation was significantly and positively associated with everyday creative ideation (β = 0.252, *p* < 0.001, 95% CI = [0.118, 0.379]). After age and gender were included as control variables in the model, neither variable was significantly associated with intrinsic motivation or everyday creative ideation (all *p* > 0.05). Figure 2 presents the standardized path coefficients after controlling for age and gender.

**Figure 2:**
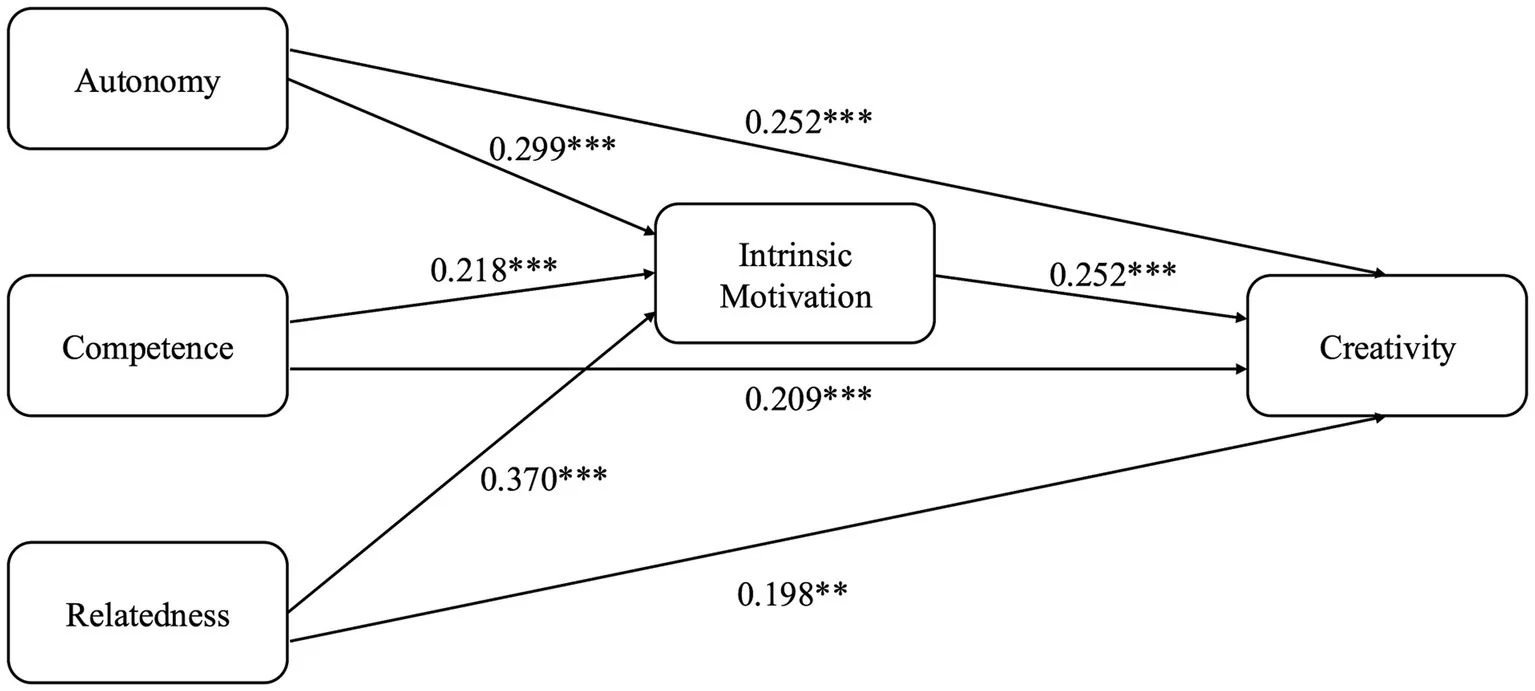
Structural model with path coefficients.

### 3.7 Mediation analysis

The indirect effects through intrinsic motivation were tested using 5,000 bootstrap resamples. As shown in Table 9, after controlling for age and gender, the indirect effect of autonomy on everyday creative ideation through intrinsic motivation was significant (indirect effect coefficient = 0.075, *p* = 0.002, 95% CI = [0.034, 0.128]), accounting for 22.94% of the total effect. In addition, the indirect effect of competence on everyday creative ideation through intrinsic motivation was also significant (indirect effect coefficient = 0.055, *p* = 0.004, 95% CI = [0.025, 0.102]), accounting for 20.83% of the total effect. The indirect effect of relatedness on everyday creative ideation through intrinsic motivation was likewise significant (indirect effect coefficient = 0.093, *p* = 0.002, 95% CI = [0.044, 0.160]), accounting for 31.96% of the total effect. The 95% confidence intervals for all three indirect paths did not include zero, indicating that autonomy, competence, and relatedness each had a significant indirect association with creativity through intrinsic motivation. Therefore, H2 was supported, but the magnitudes were modest and indicate that intrinsic motivation represented only one part of the associations.

**Table 9:** Indirect effect estimates (Bootstrap = 5,000).

Path	Effect value	*p* value	SE	95% Bootstrap CI	Result
AU → IM → CRE	0.075	0.002	0.024	[0.034, 0.128]	Supported
CO → IM → CRE	0.055	0.004	0.019	[0.025, 0.102]	Supported
RE → IM → CRE	0.093	0.002	0.029	[0.044, 0.160]	Supported

As a robustness check, the structural model was re-estimated using all 21 BPNS items. The Supplementary Table presents a side-by-side comparison of β, f^2^, R^2^, and the three specific indirect effects obtained from the 21-item and 15-item models. Across the two models, the direction and statistical significance of all hypothesized direct effects and the three specific indirect effects remained unchanged, while the β, f^2^, and R^2^ estimates were also similar. These findings suggest that item refinement had a limited influence on the structural model results.

### 3.8 Sensitivity tests

Because this study used cross-sectional data, the estimated indirect effects may be affected by potential unmeasured confounding. This study employed the mediation sensitivity analysis approach proposed by Tingley et al. (2014) to assess the robustness of the estimated indirect relationships. The analysis used the assumed correlation coefficient ρ between the residuals of the mediator and outcome models as the sensitivity parameter and examined the sensitivity of the estimated indirect effects to potential unmeasured confounding by progressively varying ρ.

As shown in Figure 3, for the AU → IM → CRE pathway, the estimated indirect effect gradually decreased as the assumed residual correlation increased and reached zero at approximately ρ = 0.42. Because the indirect effect did not disappear under lower levels of assumed residual correlation but required the residual correlation to reach approximately 0.42, the AU → IM → CRE indirect relationship demonstrated a certain degree of robustness to potential unmeasured confounding.

**Figure 3:**
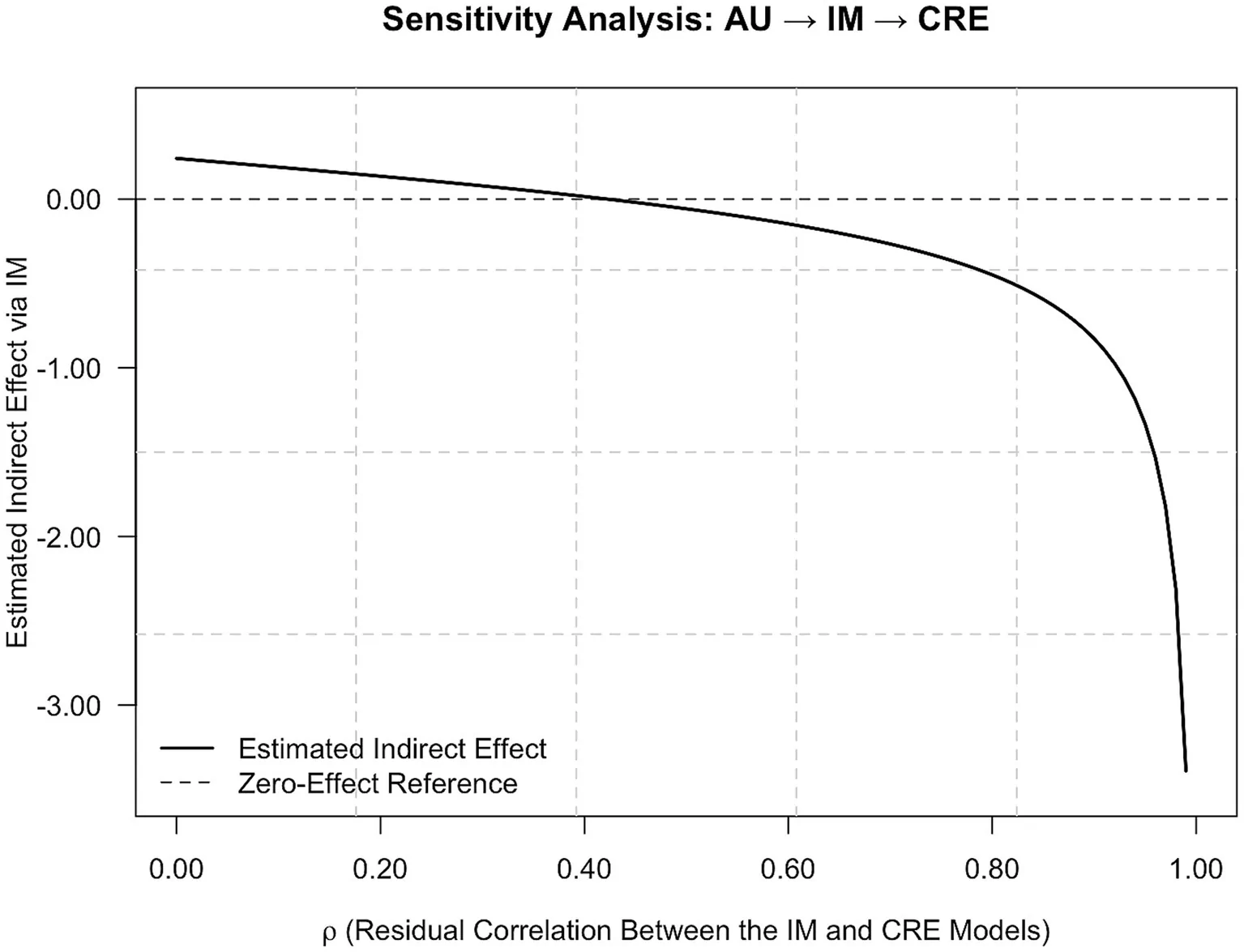
Sensitivity analysis: AU → IM → CRE.

To assess the robustness of the CO → IM → CRE indirect pathway, the sensitivity analysis results are presented in Figure 4. As the assumed residual correlation parameter ρ increased, the estimated indirect effect gradually decreased and approached zero at approximately ρ = 0.49. This means that the indirect effect would be fully attenuated only when the residual correlation between the mediator and outcome models reached approximately 0.49 or higher. The results indicate that this indirect pathway demonstrated relatively strong robustness under the specified sensitivity analysis conditions.

**Figure 4:**
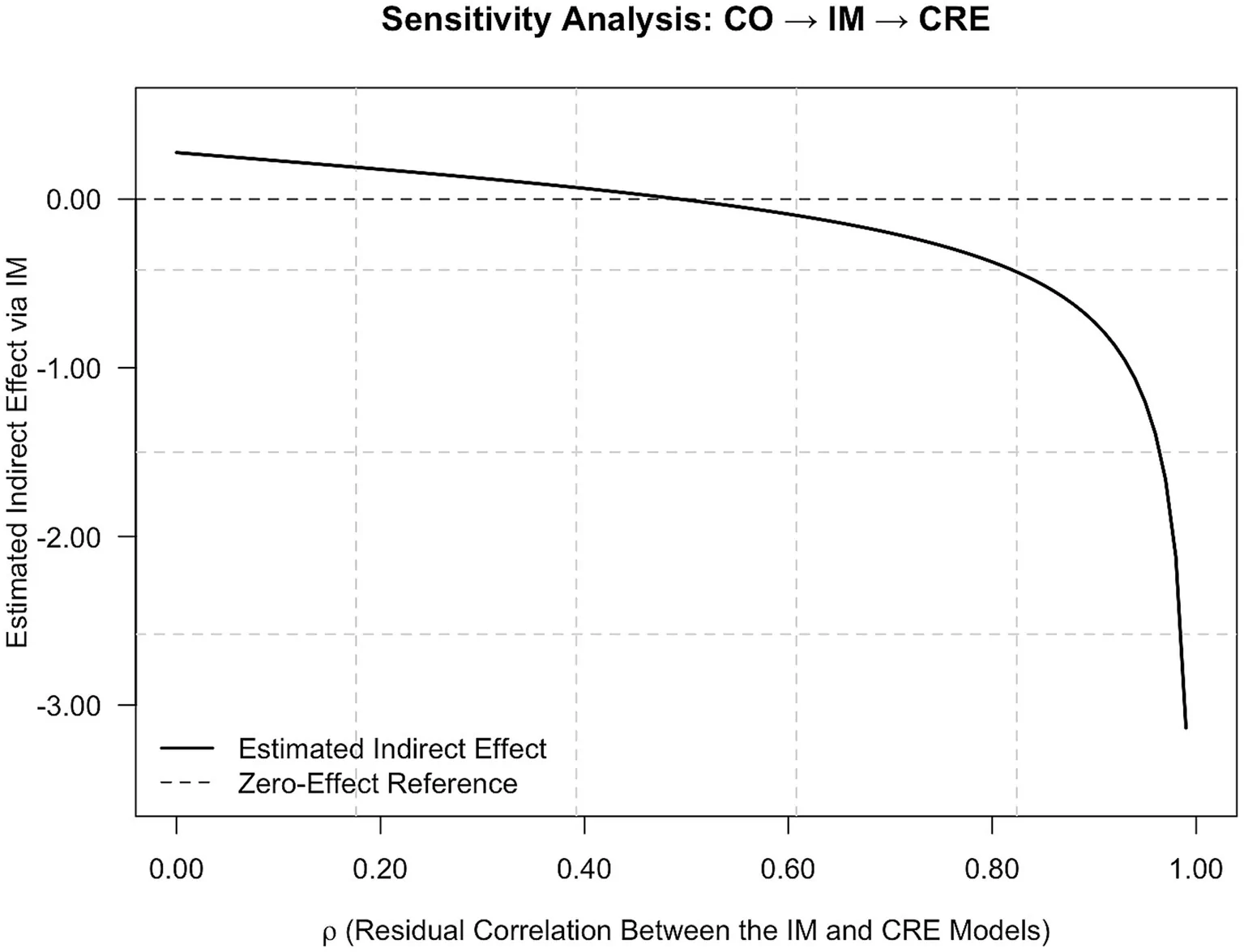
Sensitivity analysis: CO → IM → CRE.

This study further conducted a sensitivity analysis for the RE → IM → CRE pathway. As shown in Figure 5, the estimated indirect effect gradually decreased as the assumed residual correlation parameter ρ increased and approached zero when ρ reached approximately 0.43. This indicates that the indirect relationship between RE and CRE through IM retained a certain degree of robustness under assumed residual correlations below approximately 0.43.

**Figure 5:**
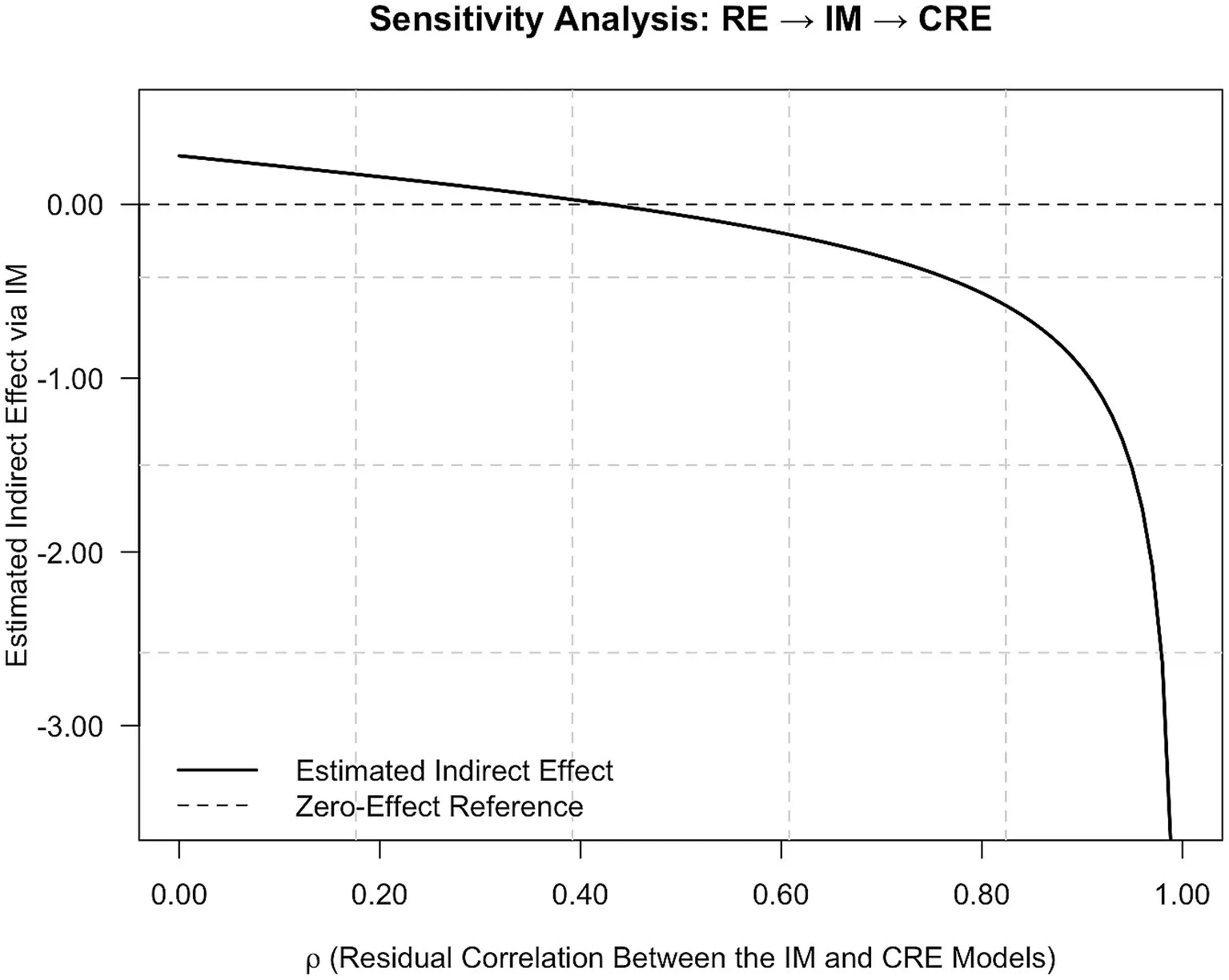
Sensitivity analysis: RE → IM → CRE.

## 4 Discussion

Using a sample of Chinese university students, this study integrated self-determination theory and the componential theory of creativity to examine the relationships of autonomy, competence, and relatedness with creativity, as reflected by self-reported ideational behavior, with a particular focus on the mediating role of intrinsic motivation. The structural equation modeling results based on cross-sectional data showed that students with higher levels of autonomy, competence, and relatedness tended to have higher levels of intrinsic motivation, while higher intrinsic motivation was significantly associated with higher levels of creative ideation. In addition, the sensitivity analyses indicated that these indirect relationships remained relatively robust to moderate levels of potential unmeasured confounding.

From a demographic perspective, this study found no statistically significant gender differences across any of the five core variables. This finding is consistent with the gender similarities hypothesis, which suggests that differences between males and females are generally small across most psychological characteristics (Hyde, 2005). Previous research has also shown that gender differences in creativity are not consistently observed and may vary depending on the specific task and domain (Taylor and Barbot, 2021). In STEM creativity, gender differences have been observed in creative self-beliefs (Vieira et al., 2025). These differences may be related to differences in creative self-efficacy and to gender stereotypes about creativity (Luo et al., 2023).

In contrast, differences were observed across age groups in competence, relatedness, and creative ideation. This finding is consistent with previous research. Bai and Guo (2026) found significant differences in basic psychological need satisfaction across different academic years. Ulger (2023) also found a significant association between students’ age and creative thinking scores, suggesting that creative thinking tendencies may be related to age or educational stage. However, the mean scores across age groups in the present study did not show a consistent upward or downward trend, indicating that age differences were more likely to reflect fluctuations between groups rather than a linear developmental pattern. Students of different ages may face different academic challenges and pressures (Yang and Yang, 2022), and such pressures are associated with their evaluations of their own competence and creativity (Yao et al., 2023). Although some variables differed across age groups, the direction and statistical significance of all hypothesized paths remained stable after controlling for age and gender.

Previous studies have shown that autonomy, competence, and relatedness are all associated with creativity. Autonomy can help students feel that they are the initiators of their own actions and act in accordance with their personal interests and values, a process that is more likely to generate novel and useful ideas (Weng et al., 2025). Messmann et al. (2022) noted that competence is essential for individuals to act confidently during complex innovation processes and to maintain motivation when facing challenges. Relatedness, in turn, can facilitate communication and collaboration and is positively associated with university students’ creativity (Singh and Aziz, 2025). These findings suggest that autonomy, competence, and relatedness are all important psychological experiences to consider when examining the psychological processes associated with intrinsic motivation and creativity among university students.

More specifically, a key finding of this study was that indirect associations between basic psychological needs and creativity through intrinsic motivation were observed for autonomy (β = 0.075), competence (β = 0.055), and relatedness (β = 0.093), with all three indirect paths reaching statistical significance. In this study, creativity was assessed through self-reported ideational behavior. Although the indirect associations were statistically distinguishable from zero, they were modest in magnitude, accounting for approximately one fifth to one third of the corresponding total associations. Theoretically, this pattern is more consistent with a partial, multi-pathway account than with one in which intrinsic motivation serves as the predominant explanatory mechanism. Intrinsic motivation may therefore represent one psychological pathway through which basic psychological need satisfaction is associated with everyday creative ideation. Because the direct associations of autonomy, competence, and relatedness with everyday creative ideation also remained statistically significant, the findings support interpreting intrinsic motivation as a complementary, rather than exclusive, pathway within the proposed theoretical model.

Undergraduate students may perceive school activities and tasks as external demands when facing pressures related to coursework, academic evaluation, and future development. In such contexts, students with higher levels of autonomy are more likely to perceive tasks as valuable and worth investing effort in and to show stronger situational interest and a greater tendency toward active participation (Johansen et al., 2025). Such experiences of autonomy are also associated with students’ everyday creativity (Mammadov et al., 2025). Students with higher levels of competence are more likely to maintain confidence and engagement when facing complex tasks. Such confidence can help strengthen intrinsic motivation and increase students’ willingness to continue refining their ideas in creative tasks (Shao et al., 2024). In socially supportive contexts, teachers’ emotional support may strengthen the positive relationship between students’ intrinsic motivation and learning engagement, which, in turn, is associated with online creativity (Wang, 2022).

The strengths of the associations between different basic psychological needs and intrinsic motivation varied to some extent. In the present study, the experience of social connection reflected by relatedness appeared to be more closely tied to specific interactional contexts during undergraduate students’ learning, particularly in learning environments where peer interaction and teacher support are important (Weng et al., 2026). Autonomy reflects the degree to which students experience self-determination in their activities and showed a relatively stable association with intrinsic motivation even in contexts characterized by both course-related pressure and external evaluation (Martinek et al., 2022). Although competence is an important foundation for sustained engagement, its role may become more evident gradually through the accumulation of task-related experience (Liem, 2022), which may help explain its numerically smaller but still statistically significant point estimate in the present sample. These findings may inform the design of practices intended to support students’ basic psychological needs and intrinsic motivation in higher education.

To further examine the stability of the mediation results under nonexperimental research conditions, this study employed the mediation sensitivity analysis approach proposed by Tingley et al. (2014). This approach assesses the sensitivity of the estimated indirect effects to potential unmeasured confounding by varying the assumed correlation between the residuals of the mediator and outcome models. The results showed that the proposed indirect pathways demonstrated a certain degree of robustness to potential unmeasured confounding.

### 4.1 Theoretical contributions and practical implications

This study makes two theoretical contributions. First, building on recent research in higher education on basic psychological needs, intrinsic motivation, and creativity, the present study examined these constructs within an integrated analytical framework. Singh and Aziz (2025) investigated the associations of autonomy, competence, and relatedness with creativity among university students in an AI-supported learning context. Weng et al. (2026) examined the relationships among personality traits, basic psychological needs, and creativity in a technology-rich university learning context and reported positive associations of autonomy and relatedness with creativity. In a more domain-specific context, Liu (2025) studied students at Chinese music colleges and found that basic psychological need satisfaction was associated with creative musical performance through pathways involving intrinsic motivation and creative self-efficacy. Against this background, the present study focused more specifically on intrinsic motivation as a potential pathway through which the three basic psychological needs may be associated with everyday creative ideation. By modeling autonomy, competence, and relatedness as distinct dimensions within a single structural model, the study simultaneously estimated the direct associations of each need with intrinsic motivation and everyday creative ideation, as well as their indirect associations with everyday creative ideation through intrinsic motivation. This approach also allowed the association patterns of the three needs to be compared within the same sample of Chinese undergraduate students. Accordingly, the findings provide a more differentiated account of how autonomy, competence, and relatedness are each associated with intrinsic motivation and everyday creative ideation, thereby extending recent research on creativity in higher education from an SDT perspective.

Second, within the Chinese educational context, students’ psychological need satisfaction and learning motivation are associated with multiple contextual factors, including the educational system, teaching practices, and the expectations of significant others (Yu et al., 2018). Perceived relatedness with teachers and peers has been associated with students’ self-regulated learning, perceived learning gains, and learning satisfaction (Zhou et al., 2021), and relatedness has also been found to have unique associations with specific learning strategies (Zhou et al., 2025). Within the Chinese undergraduate sample examined in this study, the point estimate for the association between relatedness and intrinsic motivation was numerically the largest among the three basic psychological needs, and the indirect association of relatedness with everyday creative ideation through intrinsic motivation was also numerically the largest. This pattern may reflect the relative salience of interpersonal relationships in students’ motivational experiences within Chinese higher education. Undergraduate learning and creative activities often take place through interactions with teachers and peers. Students who experience support, acceptance, and a sense of belonging in these interactions may also experience greater enjoyment when exploring and exchanging ideas. This interpretation offers one possible context-specific account of this numerical pattern. Differences in the magnitudes of the path coefficients may also reflect sample composition and unmeasured contextual factors. Moreover, the cross-sectional design does not provide a basis for ranking the three basic psychological needs in general terms.

The findings of this study may offer theoretically informed practical insights for undergraduate students, teachers, and other educational practitioners seeking to understand students’ creative ideation and its associated psychological processes in similar higher education contexts. Specifically, the findings suggest that students’ everyday creative ideation may be considered alongside their experiences of autonomy, competence, relatedness, and intrinsic motivation in creative activities. In this study, creativity primarily refers to students’ self-reported everyday ideational behavior. Therefore, the following practical implications are intended to support students’ generation of creative ideas rather than to address objective creative achievements in specific domains. At the same time, given the relatively modest magnitudes of the indirect associations observed in this study, these findings are better suited to informing cautious, theory-consistent supportive practices. First, because autonomy, competence, relatedness, and intrinsic motivation were positively associated with everyday creative ideation, students may find it useful to attend to these psychological experiences when participating in learning and creative activities. For example, in course assignments or project-based learning, students may actively participate in selecting topics, connect tasks with their personal interests, and explore different ideas during task completion. These practices are consistent with the psychological factors examined in the present study.

Second, all three basic psychological needs were significantly and positively associated with intrinsic motivation. Teachers may consider how course structures and instructional practices shape students’ experiences of basic psychological need satisfaction when designing and implementing courses. For example, teachers may provide an appropriate degree of choice, allowing students to select task topics, modes of expression, or problem-solving approaches based on their interests, thereby supporting autonomy. Teachers may also support students’ sense of competence through clear feedback, appropriately challenging tasks, and staged guidance. Meanwhile, group discussions, peer feedback, and a supportive classroom climate may facilitate students’ experiences of relatedness. These practices are theoretically consistent with the psychological processes examined in the present study and may represent potential approaches for creating more need-supportive learning environments.

Third, the findings suggest that practices intended to support students’ creative ideation may consider the psychological conditions associated with students’ participation in creative activities. According to self-determination theory, autonomy, competence, and relatedness represent distinct psychological experiences that can support internalization and engagement in activities. The present findings further suggest that intrinsic motivation may be involved in the indirect associations between these need-related experiences and everyday creative ideation. Accordingly, the practical implications should not be interpreted as suggesting that creativity can be directly enhanced through any single intervention. Instead, greater attention may be given to creating learning environments that support students’ experiences of autonomy and competence, as well as their positive connections with others. For example, universities might consider designing learning activities that offer opportunities for choice, constructive feedback, collaboration, and exchange, as these environmental features are theoretically consistent with the psychological processes examined in this study. Given the cross-sectional design, these approaches should be understood as potential supportive conditions associated with students’ creative ideation, rather than as effective interventions that this study has demonstrated can enhance such ideation.

### 4.2 Limitations and future directions

Although this study has made several contributions, several limitations should be acknowledged. First, although the directional relationships among the variables were theoretically specified based on self-determination theory and the componential theory of creativity, the causal order among these variables cannot be determined, and the possibility of reverse associations cannot be ruled out. For example, students with higher levels of everyday creative ideation may be more likely to experience interest and enjoyment in creative activities because generating and developing new ideas can itself be psychologically engaging. Repeated engagement in such activities may also strengthen students’ perceptions of competence when they feel capable of producing or refining ideas, autonomy when they experience ownership over those ideas, and relatedness when creative activity involves supportive interaction or recognition from others. These experiences may, in turn, be associated with stronger intrinsic motivation and subsequent creative ideation. Thus, the relationships among basic psychological needs, intrinsic motivation, and everyday creative ideation may be reciprocal and mutually reinforcing over time rather than operating exclusively in the direction specified in the present model. Therefore, the indirect relationships identified in this study should be understood as statistical associations consistent with the theoretical model rather than as definitive evidence for the directionality of the proposed mediation process. Future research may adopt longitudinal or cross-lagged designs to examine the temporal ordering and reciprocal associations among basic psychological needs, intrinsic motivation, and everyday creative ideation, or use instructional interventions and experimental designs to further investigate whether basic psychological needs and intrinsic motivation contribute to changes in creativity-related outcomes.

Second, this study mainly relied on self-report questionnaires. Although Harman’s single-factor test and the full collinearity assessment did not indicate substantial common method bias, some common method variance may still exist because all variables were measured by the same respondents at the same time point. This shared measurement method may have inflated the observed associations to some extent; therefore, the magnitude of the direct and indirect effects should be interpreted with caution. In addition, self-report data may still be affected by other measurement biases, such as social desirability bias. Future research could combine self-report measures with teacher ratings, peer evaluations, creativity tasks, or classroom observations to obtain more comprehensive and objective assessments. Moreover, the creativity measure used in this study was based on the Runco Ideational Behavior Scale (RIBS), which assesses individuals’ self-reported everyday ideational behavior rather than objective creative performance. Although ideational behavior represents an important aspect of creativity, it may not fully capture creativity expressed through actual products, expert evaluations, or domain-specific achievements.

Third, the sample consisted of Chinese undergraduate students recruited from five universities in one province, which may limit the generalizability of the findings to other educational levels, institution types, regions, or cultural backgrounds. Moreover, because participants’ university affiliations were not recorded to preserve anonymity, potential university-level clustering effects could not be examined. Therefore, unaccounted institutional-level clustering may have affected the precision of standard error estimates and statistical inference. Future research could use non-identifiable institutional codes while maintaining participant anonymity to assess potential university-level clustering effects and include samples from different regions and institution types to further examine the generalizability of the model.

In addition, the response format and item wording of the BPNS were modified in the present study. Specifically, the original 7-point response format was changed to a 5-point format, and all negatively worded items were rephrased as positively worded statements. Although these modifications were intended to improve the linguistic clarity of the Chinese questionnaire and reduce potential comprehension difficulties associated with negative wording, the exclusive use of positively worded items may be associated with differences in response distributions and item discrimination, as well as a greater susceptibility to acquiescent responding. Based on item-level psychometric performance and theoretical considerations, six items were subsequently removed. Although the resulting 15-item version demonstrated generally acceptable outer loadings, internal consistency reliability, and convergent validity in the present sample, both the item-refinement process and the psychometric evaluation were conducted using the same main study sample. The resulting structure may therefore be sample-specific to some extent. Moreover, the reduced item set may provide narrower coverage of the content represented in the original scale and may limit direct comparisons with studies using the original 21-item, 7-point version. The pilot test sample was also small and was used primarily to assess item clarity and comprehensibility rather than to evaluate the factor structure of the adapted scale. Accordingly, the present results should be interpreted as evidence of the internal structure of the adapted BPNS within the current sample; they do not establish its full equivalence to the original scale or its replicability across other samples. Future research should cross-validate this structure using independent samples, compare the original and adapted response formats and item wording, and further examine measurement invariance across different populations and cultural contexts.

Finally, this study mainly focused on the relationships among autonomy, competence, relatedness, intrinsic motivation, and creativity. Future research could further examine the roles of cognitive, affective, and educational contextual factors in these relationships to develop a more comprehensive understanding of the psychological processes associated with university students’ creativity.

## 5 Conclusion

Using a sample of Chinese undergraduate students, this study examined the relationships among autonomy, competence, relatedness, intrinsic motivation, and creativity as reflected by self-reported ideational behavior. The results showed that autonomy, competence, and relatedness were all significantly and positively associated with creativity, consistent with the basic propositions of self-determination theory and the componential theory of creativity. Demographic analyses indicated no significant gender differences across the five core variables, whereas significant age-group differences were observed in competence, relatedness, and creativity. However, the mean scores across age groups did not show a consistent upward or downward trend. After controlling for age and gender, the main findings remained stable. This study further employed simulation-based sensitivity analyses to examine the robustness of the indirect relationships under potential unmeasured confounding. The results indicated that all three indirect relationships showed relative robustness, although their magnitudes were modest. These findings provide empirical support for the proposed theoretical model; however, given the cross-sectional nature of the data, the observed associations should not be interpreted as causal relationships. Overall, this study provides a theoretically guided model for understanding the associations among autonomy, competence, relatedness, intrinsic motivation, and creativity among Chinese undergraduate students within the sampled higher education context.

## Data Availability

The datasets presented in this article are not readily available because the participant-level data are not publicly available because public deposition of individual responses was not included in the approved ethics protocol and informed consent materials. Requests to access the datasets should be directed to Rui Zhou, ruizhourr@gmail.com.

## Glossary

ANOVA: Analysis of Variance
AU: Autonomy
AVE: Average Variance Extracted
BPNS: Basic Psychological Need Satisfaction Scale
CI: Confidence Interval
CO: Competence
CR: Composite Reliability
CRE: Everyday Creative Ideation (RIBS)
CTC: Componential Theory of Creativity
f^2^: Effect Size
HTMT: Heterotrait–Monotrait Ratio
IM: Intrinsic Motivation
IMI: Intrinsic Motivation Inventory
PLS-SEM: Partial Least Squares Structural Equation Modeling
Q^2^: PLSpredict predictive relevance statistic
R^2^: Coefficient of Determination
RE: Relatedness
RIBS: Runco Ideational Behavior Scale
SDT: Self-Determination Theory
SPSS: Statistical Package for the Social Sciences
SRMR: Standardized Root Mean Square Residual
VIF: Variance Inflation Factor
