# Supplementary material for: Basic psychological need satisfaction and creativity among university students: the mediating role of intrinsic motivation
Source: Front Psychol. 2026 Sep 16;17:1940043. doi: 10.3389/fpsyg.2026.1940043 (PMC13624083; doi:10.3389/fpsyg.2026.1940043)
Supplement: Supplementary file 1 [file Supplementary_file_1.pdf]

Intrinsic Motivation Inventory (7 Items)

Adapted English item	Adapted Chinese item
1. I enjoyed doing the creative activity very much.	我很享受做创造性活动。
2. The creative activity was fun to do.	参与创造性活动很有趣。
3. I thought the creative activity was a fun activity.	我觉得创造性活动是有趣的活动。
4. This creative activity held my attention very well.	这项创造性活动很能吸引我的注意力。
5. I would describe the creative activity as very interesting.	我会形容创造性活动非常有趣。
6. I thought the creative activity was quite enjoyable.	我觉得创造性活动相当令人愉快。
7. While I was doing the creative activity, I was thinking about how much I enjoyed it.	在参与创造性活动时，我在想我是多么享受它。
