# Supplementary material for: Basic psychological need satisfaction and creativity among university students: the mediating role of intrinsic motivation
Source: Front Psychol. 2026 Sep 16;17:1940043. doi: 10.3389/fpsyg.2026.1940043 (PMC13624083; doi:10.3389/fpsyg.2026.1940043)
Supplement: Supplementary file 2 [file Supplementary_file_2.pdf]

Basic Psychological Need Satisfaction Scale

Autonomy: 1, 4, 8, 11, 14, 17, 20
Competence: 3, 5, 10, 13, 15, 19
Relatedness: 2, 6, 7, 9, 12, 16, 18, 21

Adapted English item	Adapted Chinese item	Initial outer loading (21-item)	Final outer loading (15-item)	Decision
1. I feel like I am free to decide for myself how to live my life.	我觉得我可以自由地决定自己如何生活。	0.722	0.749	Retained
2. I really like the people I interact with.	我真的很喜欢和我相处的人。	0.708	0.707	Retained
3. I often feel very competent.	我经常觉得自己能够很好地胜任所做的事情。	0.711	0.728	Retained
4. I feel free from pressure in my life.	我在生活中感到轻松自在。	0.594	-	Remove
5. People I know tell me I am good at what I do.	我认识的人会告诉我，我很擅长自己所做的事情。	0.723	0.764	Retained
6. I get along with people I come into contact with.	我和接触到的人相处得很好。	0.734	0.748	Retained
7. I have frequent social contact with other people.	我经常与他人进行社交互动。	0.726	0.755	Retained
8. I generally feel free to express my ideas and opinions.	我通常觉得自己可以自由地表达我的想法和意见。	0.715	0.731	Retained
9. I consider the people I regularly interact with to be my friends.	我把那些与我经常相处的人视为朋友。	0.709	0.728	Retained
10. I have been able to learn interesting new skills recently.	最近我能够学习到一些有趣的新技能。	0.637	-	Remove
11. In my daily life, I can often decide for myself what to do.	在日常生活中，我常常可以自己决定要做的事情。	0.703	0.728	Retained
12. People in my life care about me.	我身边的人关心我。	0.694	-	Remove

13. Most days I feel a sense of accomplishment from what I do.	在大多数日子里，我都能从自己所做的事情中感到成就感。	0.697	0.744	Retained
14. People I interact with on a daily basis tend to take my feelings into consideration.	每天与我相处的人往往会考虑我的感受。	0.697	0.721	Retained
15. In my life, I have many opportunities to show how capable I am.	在生活中，我有很多机会展示自己的能力。	0.713	0.769	Retained
16. There are many people I am close to.	我有很多亲近的人。	0.699	0.722	Retained
17. I feel like I can pretty much be myself in my daily situations.	我觉得我可以在日常生活中做我自己。	0.708	0.723	Retained
18. The people I interact with regularly seem to like me.	经常与我交往的人似乎很喜欢我。	0.752	0.780	Retained
19. I often feel very capable.	我常常觉得自己很有能力。	0.697	-	Remove
20. I have many opportunities to decide for myself how to do things in my daily life.	在日常生活中，我有很多机会自己决定要如何做事。	0.725	-	Remove
21. People are generally pretty friendly towards me.	总体而言，人们对我比较友好。	0.709	-	Remove

Table A, B and C. Comparison of the structural model results based on the 21-item and 15-item BPNS specifications.

Table A. Direct effects and effect sizes

Path	21-item β	21-item f^2	15-item β	15-item f^2
AU → CRE	0.266	0.072	0.252	0.070
CO → CRE	0.207	0.052	0.209	0.062
RE → CRE	0.206	0.045	0.198	0.044
AU → IM	0.285	0.086	0.299	0.106
CO → IM	0.199	0.049	0.218	0.070
RE → IM	0.390	0.187	0.370	0.177
IM → CRE	0.220	0.050	0.252	0.065
Age → CRE	0.022	0.001	0.023	0.001
Age → IM	0.010	0.000	0.010	0.000
Gender → CRE	−0.043	0.001	−0.034	0.001
Gender → IM	0.004	0.000	0.015	0.000

Table B. Explained variance

Endogenous construct	21-item R^2	15-item R^2
Intrinsic motivation	0.597	0.582
Everyday creative ideation	0.609	0.596

Table C. Specific indirect effects

Path	21-item effect	21-item 95% CI	15-item effect	15-item 95% CI
AU → IM → CRE	0.063	[0.023, 0.115]	0.075	[0.034, 0.128]
CO → IM → CRE	0.044	[0.012, 0.090]	0.055	[0.025, 0.102]
RE → IM → CRE	0.086	[0.031, 0.158]	0.093	[0.044, 0.160]
